# The application of a virtual rubber dam isolation training system in dental preclinical education

**DOI:** 10.1016/j.heliyon.2024.e34728

**Published:** 2024-07-17

**Authors:** Li Li, Xiaoli Lian, Yao Chen, Wentao Peng, Yanmei Dai, Huiru Zou

**Affiliations:** a Department of Endodontics, Tianjin Stomatological Hospital, School of Medicine, Nankai University, Tianjin, 300041, China; b Tianjin Key Laboratory of Oral and Maxillofacial Function Reconstruction,Tianjin, 300041, China

**Keywords:** Dental education, Rubber dam isolation technique, Simulation, Virtual reality

## Abstract

**Objective:**

To assess the efficacy of a virtual rubber dam isolation training system in enhancing preclinical dental education.

**Methods:**

A total of 28 Grade 4 undergraduate dental students were randomly divided into two groups: a virtual simulation priority group and a conventional phantom-head priority group. The virtual simulation priority group underwent virtual simulation training initially, followed by conventional phantom-head training. Conversely, the conventional phantom-head priority group received traditional training first, subsequently followed by virtual simulation training. Pre- and post-training theoretical knowledge examination were administered, and a practical ability assessment was conducted after the second theoretical examination. A questionnaire survey was also conducted to gauge students’ attitudes and satisfaction towards the training process.

**Results:**

After training, both groups exhibited significantly higher mean scores of theoretical knowledge examination compared to their baseline scores (*P* < 0.001). Notably, the virtual simulation priority group achieved significantly higher average scores than the conventional phantom-head priority group (*P* < 0.001,Cohen's d = 1.778). However, there was no significant difference in the mean time taken to complete the practical ability assessment between the two groups (*P>0.05,*Cohen's d = 0.19). Furthermore, the majority of students (96.4 %) strongly agreed that the virtual rubber dam isolation training enhanced their comprehension of the knowledge. 92.9 % of the students strongly agreed that the virtual training system improved their abilities of mastering the rubber dam isolation technique. Only two students (7.1 %) expressed neutrality regarding the virtual simulation effectiveness.

**Conclusions:**

This study showed that the virtual rubber dam isolation training was useful in the preclinical skills training. The integration of virtual simulation into the curriculum, particularly when prioritized over conventional methods, has shown promising results in enhancing students' theoretical knowledge and technical skills related to rubber dam isolation.

## Highlights

• The virtual rubber dam isolation training system is an innovative and effective supplemental training tool.
• It provides students with an opportunity to practice and gain experience without being in a real clinical environment.
• Such training can help students develop the skills and confidence they need to become competent dentists.

## 1 Introduction

Dental practical skill training is very important in dental education. Hands-on skill acquisition, widely recognized as the cornerstone of all skill training remains elusive for dental students due to various hardware constraints and training program limitations across different dental schools [1,2].With the development of science and technology, various virtual reality-based simulation devices have been developed and emerged in dentistry [[3], [4], [5], [6], [7], [8]].

Virtual reality is defined as a computer-generated medical simulation of a 3-dimensional image or environment with which a learner interacts in a seemingly real or physical way. Many virtual reality systems have been implemented in multiple dental programs, including educational research and clinical training [9,10]. As an effective supplemental teaching tool, virtual reality system enables the students to gain clinical experience without being in the real clinical environment [11,12]. Such technology has received an increasing attention in dental education field due to its high effectiveness and unlimited reproducibility.

Nowadays, the younger generation is more technology-oriented. These students are able to acquire skills much more rapidly through computers and internet, rather than traditional methods. We are currently in the era of digitalization where nearly everything is becoming digitalized.The evolution of technology in the modern world has made virtual reality simulation-based teaching an integral component of learning, and it is widely embraced by all participants [13].

It is well-established that rubber dam isolation technique plays a crucial role in dentistry [14,15]. This technique has emerged as a popular method in dental clinic today for its efficacy in preventing infections. It can safeguard both patients and doctors, providing a professional, safe, and comfortable medical experience. Consequently, rubber dam isolation skill training has been deemed as a fundamental component of dental education.

In this study, a virtual rubber dam isolation training system was developed to complement conventional phantom-head training. Our objective was to assess the effectiveness of this virtual simulation system and determine its optimal placement within the dental curriculum. Should it be introduced as an introductory step or utilized subsequent to the conventional phantom-head training? The findings from this investigation could yield valuable insights, potentially leading to the development of initial policy recommendations for the integration of such virtual training system into digital dental education. Future research could expand upon this system by examining its impact in the post-COVID-19 era, particularly in the context of university teaching and learning environments.

## 2 Materials and methods

### 2.1 Development of the virtual rubber dam isolation training system

**Fig. 1:**
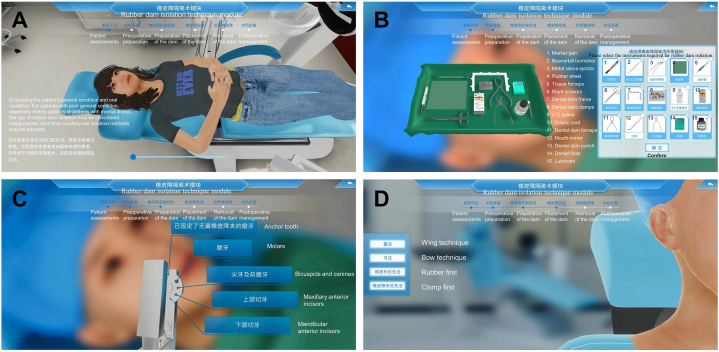
Patient assessments A.Patient assessments B. Preoperative preparation C.Preparation of the dam D. Placement of the dam.

A virtual rubber dam isolation training system was developed to meet the students’ learning needs with the collaboration of School of Artificial Intelligence, Nankai University. The system was created with Unity, a powerful software development environment designed for interactive applications, which is well known for its multiplatform three dimensional game engine with extended integration with virtual reality devices [16]. The system was very simple and clear.The application process of the virtual rubber dam isolation training system involves: 1. Patient assessments(Fig. 1 A), 2. Preoperative preparation(Fig. 1 B), 3. Preparation of the dam(Fig. 1C), 4. Placement of the dam(Fig. 1 D), 5. Removal of the dam, 6. Postoperative management. The training content was displayed three dimensionally on a computer screen. Students could log on and complete the online interactive virtual simulation training course independently. It offered virtual patients to the students, which enabled students to perform the practical procedure in a stepwise manner. The students were provided with numerous opportunities to practice all the procedures with unlimited attempts in the virtual learning environment, according to their own learning needs.

### 2.2 Experimental protocol

This study was approved by the Ethics Committee of the School of Medicine, Nankai University (No.E20230006). All experimental protocols involving human subjects were conducted following the Declaration of Helsinki (2013).

Twenty-eight dental students from Grade 4 at Nankai University were recruited and divided into two groups according to the stratified random grouping method. Initially, the students were divided into two levels by gender and then randomly assigned to either the virtual simulation priority group or the conventional phantom-head priority group. All students were willing to participate in the study and signed the informed consent form. Prior to the training, the students had already learned the principle and methods of rubber dam isolation technique in the theoretical session, but had not practiced rubber dam isolation technique either on the phantom-head or in a virtual reality environment.

The training program comprised two sessions, namely virtual rubber dam isolation training and conventional phantom-head training. In the virtual simulation priority group, the virtual simulation was carried out first and followed with conventional phantom-head training. While in the conventional phantom-head priority group, conventional training was launched first and followed with virtual simulation. The program included pre- and post-training theoretical knowledge examinations to assess the students' theoretical comprehension. Moreover,the students' practical abilities were evaluated as a critical aspect of the study. Following the second round of theoretical examination, students from both groups were tasked with using the wing technique to install rubber dams on a phantom-head. The time taken by each student to complete the installation were recorded.

**Fig. 2:**
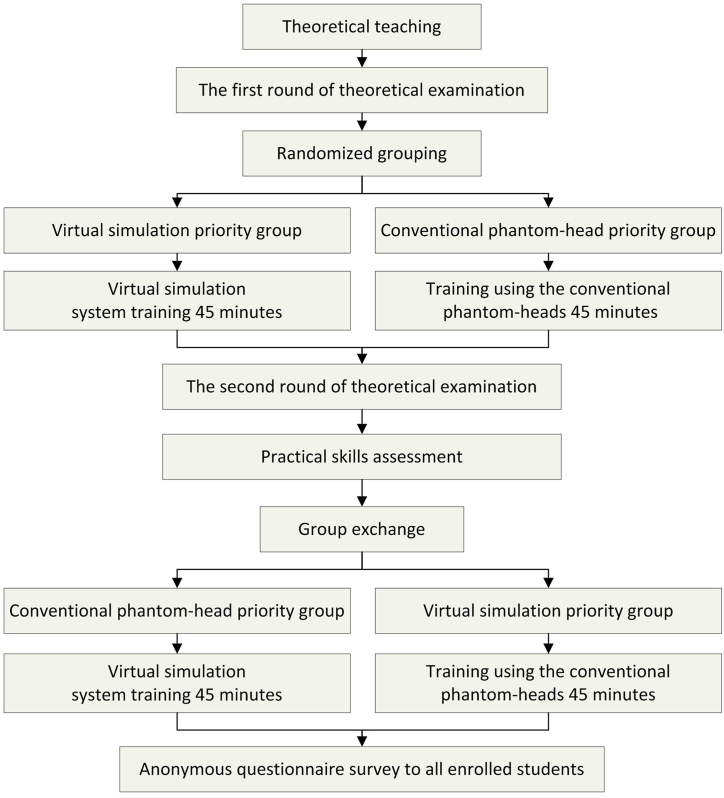
Flow diagram of the process.

During the training, the students were encouraged to ask questions and receive further verbal explanations and suggestions from the teachers. All students were asked to fill up anonymous questionnaires about their attitudes and satisfaction rates after the training. The data were then collected and analyzed(Fig. 2).

### 2.3 Questionnaire survey and statistical analysis

The questionnaire was distributed to all participants to access their attitudes towards the utilization of the virtual rubber dam isolation training system. The questionnaire used a Five-point Likert scale, ranging from 1 (strongly disagree) to 5 (strongly agree). The questionnaires were designed based on the literature review [17,18] and consultation with experts in the field. They were then pilot-tested to assess the feasibility, clarity, and relevance. Each student was able to record his or her response to each question confidentially.

All the data were collected and analyzed using SPSS statistical software (IBM SPSS Statistics V20.0, IBM Corp., Armonk, NY) and JASP software(Version 0.18.3). The Levene's test was used to assess variance homogeneity for the paired *t*-test. The level of statistical significance was set at α = 0.05. The results of the two tests were expressed by mean ± standard deviation (*x* ± *s*).

To assess the differences in the theoretical knowledge examination results, a *t*-test with effect size statistics was utilized. The effect sizes were quantified using Cohen's d statistic, where the following ranges were considered: trivial(d < 0.2), small (d = 0.20–0.49), medium (d = 0.50–0.79), and large(d > 0.79) [19]. Comparisons between two groups were conducted using the independent *t*-test, while intragroup comparisons were performed using the paired *t*-test. A *P*-value less than 0.05 was considered statistically significant.

Additionally, a separate questionnaire was developed to assess students' preferred exercise sequence for training with the virtual rubber dam isolation training system and the conventional phantom head.

## 3 Results

### 3.1 Analysis of the theoretical knowledge examination results

An independent *t*-test was conducted which indicated that there was no statistically significant difference in the mean scores of the virtual simulation priority group (76.714 ± 5.690) compared to the conventional phantom-head priority group (74.429 ± 5.034)(*P* > 0.05)(Table 1). This suggested that the students’ learning abilities were comparable before the training session.

**Table 1:** Comparison of the first round of theoretical examination results between the VSP group and the CPP group.

Groups	The first round of theoretical examination results	*t*	*p*	Cohen's d	95 % CI for the difference	
					lower	upper
**CPP Group**	74.429 ± 5.034	−1.126	0.271	0.425	−6.459	1.888
**VSP Group**	76.714 ± 5.690					

After the training, a notable improvement was observed in the mean score of the final theoretical examination for both groups (Virtual simulation priority group: *P* < 0.001, Cohen's d = 2.954 > 0.8; Conventional phantom-head priority group: *P<0.001,* Cohen's d = 2.842 > 0.8) as shown in Table 3. This highlighted that the operational training had a significant impact on enhancing the students' grasp of theoretical knowledge. Moreover, the mean scores of the virtual simulation priority group (90.643 ± 2.706) were significantly higher than those of the conventional phantom-head priority group (85.286 ± 3.292) (*P* < 0.001, Cohen's d = 1.778 > 0.8), indicating a more pronounced improvement in their theoretical understanding，as shown in Table 2.

**Table 2:** Comparison of the second round of theoretical examination results between the VSP group and the CPP group.

Groups	The second round of theoretical examination results	*t*	*p*	Cohen's d	95 % CI for the difference	
					lower	upper
**CPP Group**	85.286 ± 3.292	−4.704	<0.001	1.778	−7.698	−3.016
**VSP Group**	90.643 ± 2.706

**Table 3:** Comparison of the results from two examinations in both groups.

Groups	*t*	*p*	Cohen's d	95 % CI for the difference	
				lower	upper
**CPP Group**	−10.634	<0.001	2.842	−13.063	−8.651
**VSP Group**	−11.055	<0.001	2.954	−16.651	−11.207

Although the virtual simulation group performed better in the theoretical examination, they did not demonstrate any significant advantage in the practical skill assessment. There was no statistically significant difference in the practical time of the virtual simulation priority group (126.643s ± 13.528s) compared to the conventional phantom-head priority group (124.143s ± 12.781s)(*P* > 0.05,Cohen's d = 0.19 < 0.2)(Table 4). The improvement of practical skills requires diligent practice based on theoretical knowledge.

**Table 4:** Comparison of the duration of practical ability assessment between the VSP group and the CPP group.

Groups	The duration of practical test(sec)	*t*	*p*	Cohen's d	95 % CI for the difference	
					lower	upper
**CPP Group**	124.143 ± 12.781	−0.503	0.619	0.19	−12.724	7.724
**VSP Group**	126.643 ± 13.528					

### 3.2 Students attitude towards virtual rubber dam isolation training system

The majority of students(96.4 %, 27/28) strongly agreed that the virtual rubber dam isolation training system could enhance their in-depth understanding of the theory. 82.1 % (23/28) of students strongly agreed, and 10.7 % (3/28) agreed that the virtual system training could improve their abilities of mastering the rubber dam isolation technique. Two students (7.1 %) neither agreed nor disagreed with this statement. The detailed information is shown in Table 5.

**Table 5:** The attitude of students on the virtual rubber dam isolation training system.

Items	Strongly agree	Agree	Neutral	Disagree	Strongly disagree
The virtual rubber dam isolation training system provided clear instructions in an easy format.	26 (92.9 %)	2 (7.1 %)	0 (0 %)	0 (0 %)	0 (0 %)
The virtual rubber dam isolation training system effectively enhanced understanding of basic knowledge.	27 (96.4 %)	1 (3.6 %)	0 (0 %)	0 (0 %)	0 (0 %)
After using the virtual rubber dam isolation training system, I felt more confident about my skills.	23 (82.1 %)	3 (10.7 %)	2 (7.1 %)	0 (0 %)	0 (0 %)
The application of virtual rubber dam isolation training system was an alternative preclinical experience.	23 (82.1 %)	3 (10.7 %)	1 (3.6 %)	1 (3.6 %)	0 (0 %)

### 3.3 Students opinion about training sequence

**Fig. 3:**
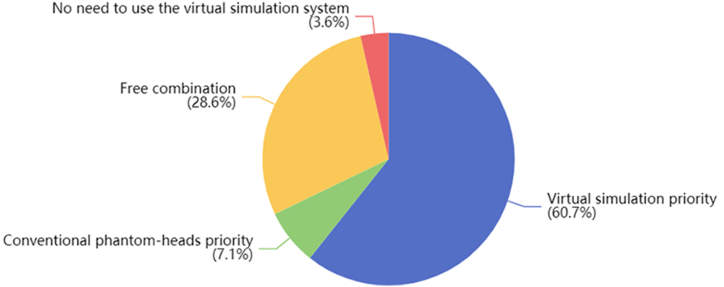
Questionnaire results of student's attitude towards training sequence.

The majority of students(96.4 %, 27/28) felt that using the virtual rubber dam isolation training system was essential. However, there were differences in the preferred sequence of exercises. According to the survey results, 60.7 % of students(17/28) preferred to start with virtual simulation and then move on to conventional training; 7.1 % of students(2/28) preferred the opposite order, and 28.6 % of students(8/28) preferred to freely combine virtual simulation and conventional training(Fig. 3).

The results of the questionnaire survey could be summarized as follows: The virtual rubber dam isolation training system received positive feedback from students. It was well-accepted and enjoyed by the participants. Students tended to prefer combining the virtual systems with conventional phantom-head training. The majority of students held a positive attitude towards the virtual system.

## 4 Discussion

Preclinical dental skill training is very essential in dental education.It serves as a foundation to build students skills about the clinical procedures and techniques that students will apply in real-world settings. As an important part of dental training, simulation has a long history.The use of simulation devices, such as mannequins or phantom heads, allow students to hone their dental skills in a controlled and risk-free environment.However, there are time and space limits considering about students’ practice on phantom heads. Other methods or facilities need to be developed to assist preclinical training.

With the development of computer technology and virtual technology, many virtual simulation systems have been developed and applied to dental education [[3], [4], [5], [6], [7]].The virtual training system uses computer-based simulation to create an immersive and interactive learning environment that allows students to practice their skills in a safe and controlled environment. It has been applied in the preclinical training of oral surgery, endodontics, oral histopathology, dental implants and periodontal disease [[20], [21], [22], [23], [24]]. Simulation software provides identical clinical simulations for all students. Virtual-reality simulation also provides support for the treatment planning of periodontal disease, implants as well as the orthognathic problems [2]. Virtual reality in restorative dentistry and endodontics creates a digital environment to practice cavity preparation, caries evacuation, and light-curing techniques. Such virtual systems are beneficial for improving the clinical skills of students [25,26].

Compared to the classical training program, virtual simulation systems have such advantages, first, the virtual system allows repeatable and accurate training. Repeated exercises are vital for students in achieving the required mastery level [27,28]. Virtual-reality technologies can be used at home or anywhere once necessary, which extending further the advantages of this type of training.Second, virtual system provides students with the opportunity to practice independently, without the direct supervision of a teacher or mentor. Such training is helpful in building the responsibility and self-sufficiency of students. Finally, virtual system generates no medical waste and requires fewer consumables [13].

In this study, we developed a virtual rubber dam isolation training system and conducted a comparison study about the effects and sequences of this virtual rubber dam isolation training system and conventional phantom-heads training in dental preclinical education. Phantom-heads training have been considered as standard pedagogical tools in rubber dam isolation technique teaching [29].These models have played important roles in helping students master basic skills, but they also have their limitations. It is boring to practice over and over again. Without timely feedback, students do not know if their actions are right or wrong. With the assistance of virtual rubber dam isolation system, students can clearly grasp the operation procedure. Any errors will be immediately pointed out by the system and then corrected by students. The results suggested that the virtual rubber dam isolation training system were highly recommended by the students.This statement provided an interesting perspective on the potential of virtual reality technology in education, specifically in dental education. In our study, 92.9 %(26/28) of the students believed that the virtual rubber dam isolation training system could be served as a valid preclinical experience. The virtual simulation system could not only immerse students in a highly engaging operational environment but also feature comprehensive prompts and operational guidance. It made preclinical training more convenient and accessible, providing a platform for students to practice their skills in a controlled and risk-free environment. The theoretical examination results of both groups after the whole training were one of the evidence.

Nowadays, students who have grown up in the digital era, with easy access to smartphones, tablets, and laptops, tend to be more comfortable and familiar with technology. This means they are more likely to embrace and seek out opportunities to learn through digital platforms. Virtual simulation training systems can provide a convenient and engaging platform for students to access knowledge and skills. They offer flexibility and accessibility, allowing students to learn at their own pace and from anywhere with an internet connection. This flexibility can be particularly appealing to today's students, who often have busy schedules and prefer to learn in non-traditional settings. The interactive and immersive nature of virtual simulation systems can also enhance students' engagement and motivation to learn. These systems provide a controlled and risk-free environment where students can practice and refine their skills without the pressure or risks associated with real clinical settings. The comprehensive guidance and feedback features can further support students in their learning process, ensuring they are developing the necessary knowledge and skills effectively. Therefore, students tend to be more open to learning through virtual platforms.

Interestingly, the improvement in the conventional phantom-head priority group was even more significant than the virtual simulation priority group. This indicated that the order of virtual simulation training and conventional phantom head training was also important. Some studies suggested that starting with virtual simulation training may provide students with a foundational understanding of the procedures involved before practice on the phantom head. This approach can help them build confidence and familiarity with the techniques before they face with the real tasks [[30], [31], [32], [33]]. Some studies recommended starting with phantom head training. They believed that phantom head training may allow students to develop basic manual dexterity and gross real impressions before touching virtual simulation techniques. This approach can help them have a solid grasp of the fundamental techniques before enhancing through virtual reality. In general, it is essential to note that the specific order may vary depending on the individual's learning style, the goals of the training program, and the type of skills being taught. It is important to consider the benefits of each approach and how they can best complement one another to achieve the desired learning outcomes ultimately.

The pressure on educators to assist students in achieving the requisite skill levels in a short time frame can indeed be intense. To address this challenge, there is a need for universities to turn to technology based teaching and learning software to enhance students’ learning [8]. Educators can utilize virtual simulation techniques that minimize the time required for students to gain the necessary skills while still ensuring they have a solid foundation in the subject matter. Given the benefits of virtual reality-based training, it is essential that virtual reality sessions are integrated into the dental curriculum as a bridging element between theoretical and practical sessions [34].It is also important to recognize that each student has different learning styles and abilities, and not all will learn at the same pace. Educators should tailor their teaching methods to cater to the diverse needs of their students, ensuring that everyone has the opportunity to develop their skills effectively.

## 5 Conclusion

Within the limitations of the present study, the results clearly suggested that the virtual rubber dam isolation training system was very useful for preclinical skill training. Meanwhile, further experiments were also needed to explore the long-term retention of skills acquired through these training.

## Fundings

This work was supported by the Ministry of Education, P.R.China [grant number ZJXF2022003]; Science and Technology Program of Tianjin [grant numbers 22KPXMRC00040,22JCYBJC01240]; Tianjin Key Medical Discipline(Specialty)Construction Project [grant number TJYXZDXK-078D]; Tianjin Municipal Health Commission Program ZC20070 and the Medical Education Research Project of Medical Education Branch of Chinese Medical Association[grant number 2023B173].

## Data availability statement

Data associated with this study are included in this article and its supplementary files.

## CRediT authorship contribution statement

**Li Li:** Writing – review & editing, Writing – original draft, Validation, Methodology, Investigation, Funding acquisition, Conceptualization. **Xiaoli Lian:** Writing – review & editing, Writing – original draft, Validation, Software, Methodology, Investigation, Formal analysis, Conceptualization. **Yao Chen:** Writing – review & editing, Writing – original draft, Validation, Methodology, Investigation, Data curation. **Wentao Peng:** Writing – review & editing, Writing – original draft, Validation, Methodology, Investigation, Data curation. **Yanmei Dai:** Writing – review & editing, Writing – original draft, Visualization, Supervision, Methodology, Investigation, Funding acquisition, Data curation, Conceptualization. **Huiru Zou:** Writing – review & editing, Writing – original draft, Supervision, Software, Methodology, Investigation, Funding acquisition, Formal analysis, Data curation, Conceptualization.

## Declaration of competing interest

The authors declare that they have no known competing financial interests or personal relationships that could have appeared to influence the work reported in this paper.
