# Supplementary material for: The application of a virtual rubber dam isolation training system in dental preclinical education
Source: Heliyon. 2024 Jul 17;10(14):e34728. doi: 10.1016/j.heliyon.2024.e34728 (PMC11734150; doi:10.1016/j.heliyon.2024.e34728)
Supplement: Multimedia component 1 [file mmc1.docx]

**Questionnaire on The application of a virtual rubber dam isolation training system**

In order to gain a deeper understanding of the application effect of the virtual rubber dam isolation training system in the teaching of Operative dentistry and endodontics, we have designed this questionnaire for investigation. Please tick the corresponding option with a "√". If you have any suggestions or opinions, please feel free to write them in the blank space provided. Your feedback is of utmost importance to us in making informed decisions, and we sincerely appreciate your support and cooperation. Thank you!

1. **The virtual rubber dam isolation training system provided clear instructions in an easy format.**
2. Strongly agree ( ) B. Agree ( ) C. Neutral ( ) D. Disagree ( ) E. Strongly disagree ( )
3. **The virtual rubber dam isolation training system effectively enhanced understanding of basic knowledge.**

A.Strongly agree ( ) B. Agree ( ) C.Neutral ( ) D. Disagree ( ) E. Strongly disagree ( )

1. **After using the virtual rubber dam isolation training system, I felt more confident about my skills.**
2. Strongly agree ( ) B. Agree ( ) C.Neutral ( ) D. Disagree ( ) E. Strongly disagree ( )
3. **The application of virtual rubber dam isolation training system was an alternative preclinical experience.**

A.Strongly agree ( ) B. Agree ( ) C.Neutral ( ) D. Disagree ( ) E. Strongly disagree ( )

1. **What was your preferred exercise sequence for training with the virtual rubber dam isolation training system and the conventional phantom-heads.**
2. The virtual simulation priority. ( )
3. The conventional phantom-head priority ( )
4. There is no need to use the virtual simulation system ( )
5. Free combination ( )

**6. Any suggestions?**
